# Ion-Selective Properties of Armbrusterite Mineral and the Prospects for Its Laboratory Synthesis

**DOI:** 10.3390/molecules30224385

**Published:** 2025-11-13

**Authors:** Darya Gryaznova, Taras Panikorovskii, Galina Kalashnikova, Ayya Bazai, Margarita Glazunova, Ekaterina Selivanova, Alevtina Gosteva, Victor Yakovenchuk

**Affiliations:** 1 Nanomaterials Research Centre, Kola Science Centre, Russian Academy of Sciences, 184209 Apatity, Russia; t.panikorovskii@ksc.ru (T.P.); g.kalashnikova@ksc.ru (G.K.); a.bazai@ksc.ru (A.B.); m.glazunova@ksc.ru (M.G.); e.selivanova@ksc.ru (E.S.); v.yakovenchuk@ksc.ru (V.Y.); 2 Geological Institute of the Kola Science Centre, Russian Academy of Sciences, 184209 Apatity, Russia; 3 I.V. Tananaev Institute of Chemistry and Technology of Rare Elements and Mineral Raw Materials, Kola Science Centre, Russian Academy of Sciences, 184209 Apatity, Russia; a.gosteva@ksc.ru

**Keywords:** armbrusterite, heterophyllosilicate, ion exchange, Cs-selective sorbent, birnessite, serandite, hydrothermal synthesis

## Abstract

In this paper, results of studying the properties of armbrusterite, a natural heterophyllosilicate, are presented. Due to its crystal structure consisting of parallel HOH-type sheets separated by a network of large-diameter (4–6 Å) channels, this mineral is of interest as a prototype for producing novel compounds to be used as sorbents. The studies were conducted by X-ray powder diffraction and X-ray single crystal diffraction analyses, electron microscopy, and IR spectroscopy. The natural sample is shown to be able to selectively extract monovalent cations from the solution of complex composition consisting of mono- and divalent salts. Furthermore, the preliminary results of hydrothermal synthesis of an analog of this mineral are reported. It is demonstrated that the mineral can be produced under milder conditions than the known methods for synthesizing heterophyllosilicates using platinum capsules (Tuttle bombs) at 380–450 °C.

## 1. Introduction

The rapid pace of industrial development in the modern world is accompanied by an increasingly harmful impact on the environment [1,2]. Therefore, there is a constantly growing need for innovative and efficient solutions to mitigate the negative impacts of the new stages of production lines that are mostly related to the mining and mineral processing industries, precious metal extraction, paint and coating production technologies, and radioactive waste management [3]. Sorption-based purification methods are widely employed for reducing pollutant concentrations in industrial by-products to the maximum permissible levels [4,5]. The sorption technologies used for extracting toxic and heavy metals from gaseous and liquid media [6] are highly efficient in addressing these challenges, particularly within the low-concentration range [7]. Natural or nature-inspired synthetic sorbents exhibiting selectivity toward specific elements are often used for this purpose [8,9].

Heterophyllosilicate compounds having a layered crystal structure can be of interest to perform a targeted search and lay the groundwork for expanding the list of available sorbents [10,11,12,13]. A feature of these compounds is that they crystallize as a “layered cake”, which typically contributes to formation of interplanar channels. In turn, this structure allows the compounds to exhibit good sorption properties and opens up the possibility for using them to model hybrid organometallic materials for pillaring [14] and membrane technologies. In modern materials science, engineering of individual 2D nanolayers is especially relevant for technological applications in designing organoclay hybrid materials and columnar structures [15,16].

Materials synthesized under laboratory conditions are commonly utilized in such studies. Nonetheless, the detailed mechanism of action of a material can often be elucidated only using natural objects because they have a more ordered crystal structure, making high-quality data collection feasible. It is especially relevant for a new mineral that does not have a known synthetic analog yet. In these cases, studies and experiments using natural samples allow one to preliminarily identify interesting chemical and physical properties of a new substance, as well as determine its practical prospects and the need to synthesize an analog. Furthermore, this format of studies enables broadening the range of promising materials exhibiting strict selectivity with respect to certain elements [17,18].

This paper focuses on the sorption capacity of armbrusterite (K_5_Na_6_Mn^3+^Mn^2+^_14_[Si_9_O_22_]_4_(OH)_10_∙4H_2_O), a rare and poorly studied mineral belonging to the heterophyllosilicate class, with respect to monovalent metal cations Cs^+^ and Rb^+^. This mineral was also shown to be stable in aggressive environments (solutions of nitric and hydrochloric acids used as an example).

The investigated mineral was first discovered at the Kukisvumchorr deposit (the Khibiny alkaline massif, Kola Peninsula, Russia) by V.N. Yakovenchuk, G.Yu. Ivanyuk, and Ya.A. Pakhomovsky (Geological Institute, Kola Science Centre of the Russian Academy of Science) in 2005. The mineral was found in a cancrinite–aegirine–microcline vein within ijolite–urtite. The mineral is associated with raite, lamprophyllite, sphalerite, galena, aegirine, manganoneptunite, cancrinite, and calcite. Armbrusterite occurs as fractured bent crystals and spherulites. The mineral is dark reddish-brown and semi-transparent [19]. Known to be a heterophyllosilicate not containing titanium, this mineral deserves special attention as it contains a network of channels with diameters ranging from 4 to 6.3 Å and can be regarded as a molecular sieve similar to zeolite-like materials [20,21].

## 2. Materials and Methods

### 2.1. Materials and Chemicals

Ion-exchange experiments were conducted using specimens of mineral crystals from the personal collection of V.N. Yakovenchuk, the leading researcher of the Nanomaterials Science Centre, Kola Science Centre, Russian Academy of Sciences: natural Mn hydrous heterophyllosilicate armbrusterite, ideally K_5_Na_6_Mn^3+^Mn^2+^_14_[Si_9_O_22_]_4_(OH)_10_·4H_2_O, found in a thin cancrinite-aegirine-microcline vein within urtite at Mt. Kukisvumchorr from the Khibiny alkaline massif, Kola Peninsula, Russia [19].

**Figure 1:**
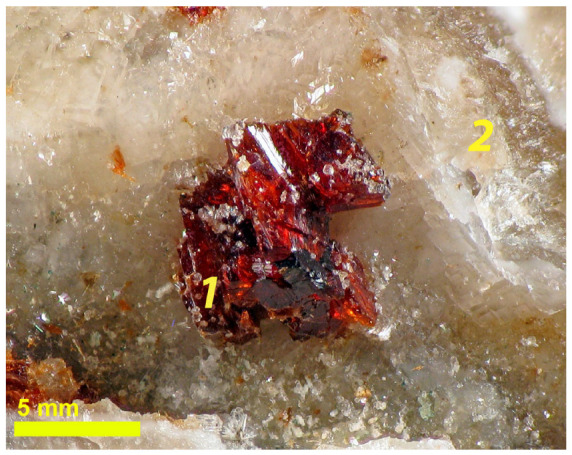
A sample of natural armbrusterite (1) on calcite (2) (the photo was provided by G.Yu. Ivanyuk).

The mineral is an aggregate having a mosaic structure and formed by crystallites sized up to 20 µm (Figure 1).

The model solutions of salts for conducting ion-exchange experiments were prepared using reagents CsCl, RbCl, SrCl_2_, and CdCl_2_ of chemically pure grade (NevaReaktiv, St. Petersburg, Russia); the compounds were synthesized using Na_2_SiO_3_∙5H_2_O, MnSO_4_∙5H_2_O, KCl, NaOH, KOH, and KBr of chemically pure grade (NevaReaktiv, St. Petersburg, Russia) and distilled water as a solvent. The experiment utilized 1 M HCl and HNO_3_ to determine the mineral’s stability in acidic media.

### 2.2. Ion-Exchange Experiments with Natural Samples

For conducting ion-exchange experiments and determining stability of the mineral in acid solutions, armbrusterite samples sized 1–2 mm were placed into model 0.1 M solutions of CsCl, RbCl, SrCl_2_, and CdCl_2_ and 0.1 M solution of complex chemical composition containing CsCl, RbCl, SrCl_2_, and CdCl_2_ salts. The mineral’s stability in acidic media was assessed by treating the samples with solutions of 1 M HCl and HNO_3_. The experiments were conducted under static conditions at room temperature, under occasional stirring, for 3 days. After the experiment, the samples were washed with distilled water and air-dried.

### 2.3. Hydrothermal Synthesis

The experiments on producing a synthetic analog of the mineral were conducted under hydrothermal conditions by mixing the initial components of the reaction mixture (the ratios between the components are listed in Table 8). The green mixture was dissolved in distilled water, and the resulting solutions were placed into autoclaves. Synthesis was carried out in 20 and 100 mL stainless autoclaves with PTFE and PPL liners (TOPH, Ningbo, China). The synthesis duration was varied from 4 to 10 days. The synthesis temperature was selected to lie within the range of 200–270 °C ensuring the maximum allowable temperature mode for the PTFE and PPL coatings. The reagents for the experiments and synthesis were weighed on an ED224SRCE analytical balance (Sartorius, Göttingen, Germany).

### 2.4. XRD Analysis

The powder X-ray diffraction data were collected using a MiniFlex 600 X-ray powder diffractometer (Rigaku, Akishima, Japan) in the 2Ɵ range of 3–70° with scanning steps of 0.02°. The normal-focus Cu X-ray tube was operated at 40 kV and 15 mA.

### 2.5. *Single-Crystal X-Ray Structure Analysis*

A single-crystal X-ray diffraction study of Cs-exchanged armbrusterite (Arm:Cs) was conducted using an XtaLAB Synergy-S diffractometer (Rigaku Corp., Japan) equipped with a HyPix-6000HE hybrid photon counting detector at the Center of the Collective Use of Equipment, Kola Science Centre. The data were integrated and corrected using the CrysAlisPro v. 41.104a [22] software package, which was also used to apply an empirical absorption correction using spherical harmonics, as implemented in the SCALE3 ABSPACK scaling algorithm. The SHELXL program from 13 March 2018 https://shelx.uni-goettingen.de [23] was used for crystal structure refinement. The structure of Cs-exchanged armbrusterite (Arm:Cs) was solved in the C2/*m* space group and refined to R_1_ = 0.13 for 4019 independent reflections with Fo > 4σ(F_o_). The crystal structure was refined according to atom names and unit cell setting with the previous structure model [16]. Ion-exchange crystals were used to determine armbrusturite intergrowths along (110), which affect the residual density peaks at 2.98 e^−^ Å^−3^ within a distance of 1.008 Å to the Mn5 site. Nevertheless, the structural model allows us to clearly define the Mn^3+^ position, whose location coincides with the previous model of unchanged armbrusterite [16], confirming the plausibility of the model obtained by us. The ECoN21 software v.1.8 was used for bond valence sum calculations [24]. The SCXRD data are deposited in the CCDC under entry No. 2415711. Crystal data, data collection information and structure refinement details are summarized in Table 1; Table 2 and Table 3 list the atomic coordinates and selected interatomic distances, respectively. The anisotropic parameters of atomic displacements are presented in Table 4.

### 2.6. IR Analysis

IR spectroscopy analysis in KBr pellets was conducted using an FT-803 Fourier transform spectrometer (Simeks Research and Production Company, Novosibirsk, Russia, 2022) within the range of 4000–400 cm^−1^ (resolution, 4 cm^−1^) at room temperature. To reduce the signal-to-noise ratio, the number of scans was 16. The spectra were analyzed using the algorithms implemented in the OriginPro 8.1 software package.

### 2.7. Chemical Composition

The morphological characterization of the synthetic powders of natural and synthetic samples was carried out using a LEO-1450 scanning electron microscope (Carl Zeiss Microscopy, Oberkochen, Germany). The chemical composition of the synthetic products was studied using a LEO-1450 equipped with X-ray energy dispersive system AZtec with detector ULTIM MAX 100 (Oxford Instruments, Oxfordshire, UK) at 20 kV, 500–1000 pA, 1–3 µm beam diameter (Geological Institute of the Federal Research Centre “Kola Science Centre of the Russian Academy of Sciences”). The water content in the samples was not measured, but calculated based on the lack of analysis sum.

## 3. Results

### 3.1. Acidic Treatment, Ion Exchange and Chemical Composition

**Figure 2:**
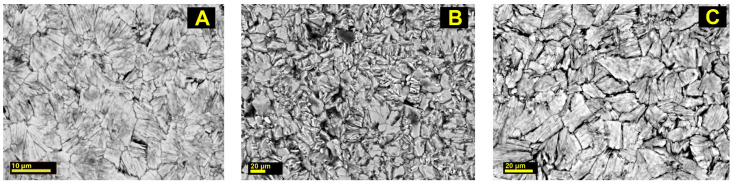
SEM images of the polished sections of the unmodified armbrusterite (**A**) and armbrusterite sample treated with 1 M acids HCl (**B**) and 1 M HNO_3_ (**C**).

The experiments in the work [25] with 5 M HCl and HNO_3_ solutions of acids demonstrated that the appearance of the initial crystals remained unchanged; the sample broke into small pieces when slightly pressed with forceps and became opalescent. After being treated with 5 M solutions, the samples were too soft and suitable for neither conducting single-crystal studies nor preparing polished specimens to identify the chemical composition [25]. Therefore, these samples were withdrawn from the experimental series. In this work, 1 M solutions of these acids were used. The samples were characterized by unchanged shape of initial crystals, became looser and lighter colored. In both cases, the appearance of the sections of armbrusterite aggregates demonstrates that the mineral can retain its crystallinity regardless of changes in hardness (Figure 2).

Treatment with acid solutions yields mineral modification containing no potassium cations as additionally proved by single-crystal studies. There eventually remains the silicon–manganese framework, which can subsequently be investigated as a platform for doping with new elements and designing materials with predictable properties.

**Figure 3:**
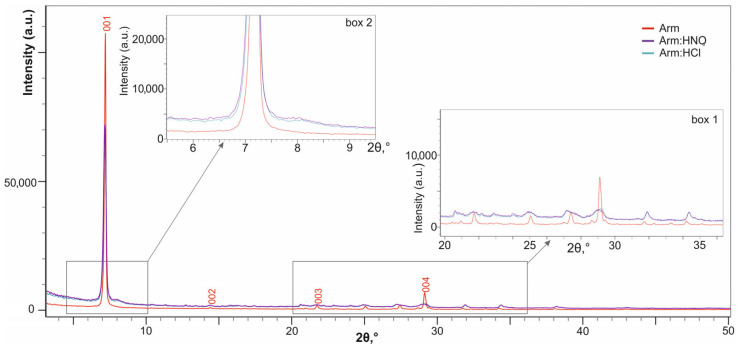
XRD patterns of initial armbrusterite and products of its treatment with acids. Box 1 and 2 are enlarged graph fragments.

A comparison of the XRD analysis data for Arm:HCl and Arm:HNO_3_ powders with the initial armbrusterite Arm (Figure 3) shows that the position of the diffraction peaks (or reflections) remain practically unchanged. This indicates that the treated samples are still crystalline armbrusterite, or most likely contain its relics. At the same time, the following observations are noted:

1. a decrease in the intensity of basal reflections (for the (004) reflection, this is clearly seen in box 1 of Figure 3). These changes are associated with texture modification, a less expressed foliation of the product compared to the original one.
2. blurring/broadening of reflections to the right up to the formation of a shoulder (the shoulder corresponding to d ≈ 11 Å is clearly visible in box 2 of Figure 3). This effect is associated with the formation of small clusters of matter with reduced interplanar distances, ≈ 11.0 Å versus ≈ 12.3 Å for the Arm, along with the relics of the latter.
3. The XRD patterns of the treated products are identical, which means that different acids affect armbrusterite in a similar way.

Table 5 lists the chemical composition of the polished sections of the samples treated with acids. The reported data indicate that while the content of silicon forming the anionic framework remains unchanged, the contents of the remaining elements decrease. Potassium is eliminated from the crystal structure of the mineral most easily; elimination of sodium is noticeable only after the mineral was treated with a hydrochloric acid solution. Manganese is eliminated partially in both solutions of acids.

The empirical formulas of armbrusterite and Arm:HCl calculated for 36 Si atoms can be written as follows:
ArmNa_5.27_K_8.60_Mn^3+^_1.11_(Mn^2+^_13.96_Fe_0.41_Ca_0.14_Al_0.09_Ti_0.07_Zn_0.08_)_∑14.89_[Si_9_O_22_]_4_(OH)_7.65_∙16H_2_OArm:HClNa_1.08_K_0.44_Mn^3+^_0.75_(Mn^2+^_5.44_Ca_0.07_Ti_0.05_Fe_0.17_)_∑5.73_[Si_9_O_22_]_4_∙9.4H_2_O

The empirical formula of the sample treated with 1 M solution of HNO_3_ acid was not determined due to the destruction of its crystalline structure.

**Figure 4:**
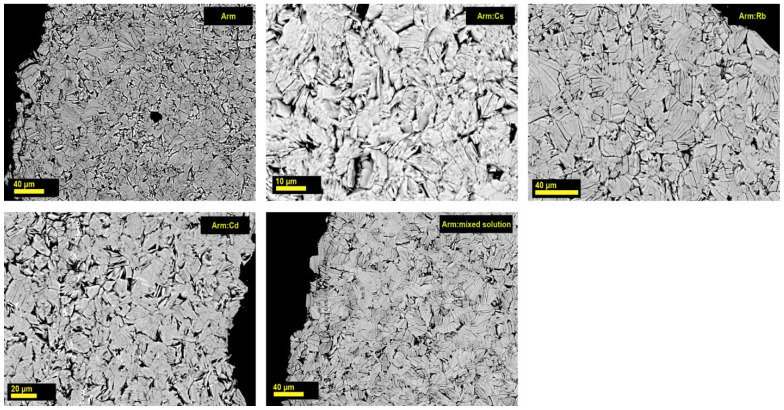
SEM images of the polished sections of crystals of the unmodified natural armbrusterite (Arm), its ion-exchanged forms Arm:Cs, Arm:Rb, and Arm:Cd, and the mineral after its contact with a multicomponent solution (Arm + mixed solution).

It was found that after treating the armbrusterite samples with solutions of salts, the appearance of the initial crystals in the sample, its hardness and color were unchanged; no visible alterations in the morphology were observed (Figure 4). Analyses with exchangeable cation content below 0.35 wt% omitted due to the impracticality of considering such samples as promising sorbents.

**Figure 5:**
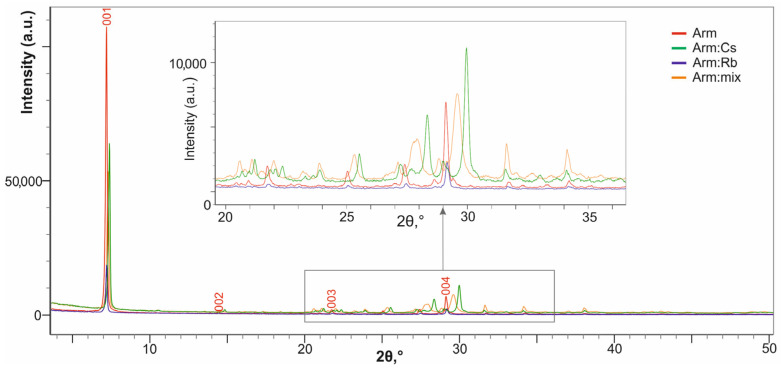
XRD patterns of armbrusterite (Arm) and its ion-exchange forms after contact with solutions of salts: Arm:Cs, Arm:Rb and Arm:mix.

These observations are additionally supported by the X-ray powder diffraction analysis of the samples (Figure 5).

The XRD patterns of the exchange products show that the basal reflections are shifted to higher angles relative to the initial armbrusterite Arm (Figure 5). As shown in the inset (Box) of Figure 5, the (004) reflection for Arm:Cs is shifted to the right by 0.9°, corresponding to a decrease in the interplanar spacing by 0.09 Å. This observation corresponds to the data of the Arm:Cs single crystal study (see below), indicating a significant decrease from parameter *c* to 12.926 Å compared to 13.489 Å for the initial armbrusterite with minimal changes in parameters *a* and *b*.

The shift in the Arm:Rb spectrum is quite small, reflecting a low degree of K^+^ substitution by Rb^+^ compared to Arm:Cs (see also Table 6).

In addition to the shift, reflection broadening is also observed for the Arm:mix sample, which is expected due to the non-uniform distribution of substituting ions within the base crystal structure.

The strongest reflections of the XRD patterns for armbrusterite (Arm) and its ion-exchange forms, Arm:Cs, Arm:Rb, Arm:Cd and Arm:mix, are listed in Table 6.

Quantitative analysis of the composition of the polished crystal section was conducted for the ion-exchanged armbrusterite samples with high concentrations of Cs^+^, Rb^+^, Cd^2+^, cations. The chemical composition of the ion-exchanged samples is listed in Table 7.

The empirical formulas of the ion-exchanged forms of armbrusterite calculated for 36 Si atoms can be written as follows:
Arm:RbNa_3.93_K_3.52_Rb_2.23_Mn^3+^_1.09_(Mn^2+^_14.00_Fe_0.42_Ca_0.13_Al_0.13_Ti_0.05_)_∑14.73_[Si_9_O_22_]_4_(OH)_5.57_∙16H_2_OArm:mixed solutionNa_3.17_K_1.0_Rb_0.23_Cs_2.42_Mn^3+^_1.06_(Mn^2+^_14.54_Fe_0.43_Ca_0.17_Al_0.16_Ti_0.08_Cd_0.03_)_∑15.41_[Si_9_O_22_]_4_(OH)_4.88_∙8.3H_2_OArm:CsNa_5.02_K_0.28_Cs_4.05_Mn^3+^_1.06_(Mn^2+^_13.46_Fe_0.38_Ca^2+^_0.12_)_∑13.96_[Si_9_O_22_]_4_(OH)_4.22_∙0.84H_2_O

The results of analyzing the distribution of sorbed elements in the unmodified mineral and its ion-exchanged forms proved that almost all the potassium ions within armbrusterite were exchanged for cesium and rubidium ions, especially in the solutions of salts containing a single monovalent metal cation. Non-significant exchange of K^+^ for Cd^2+^ cations takes place in the mineral samples treated with a solution of salt containing bivalent cations. Ion exchange from the multicomponent solution demonstrates that the mineral is selective exclusively with respect to Cs^+^ cations.

### 3.2. Single-Crystal X-Ray Diffraction

**Figure 6:**
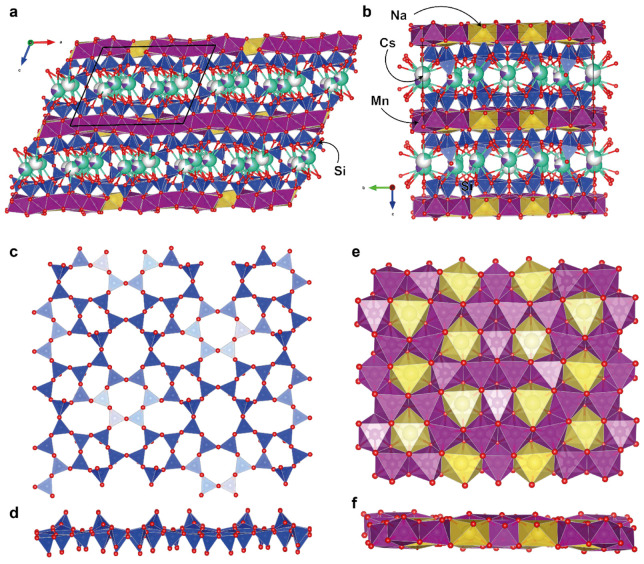
The crystal structure of Cs-substituted armbrusterite: a projection of the overall structure along [010] (**a**) and [011] (**b**); projections of the silicate [Si_9_O_22_] layer (**c**,**d**) and Na-Mn layer [Mn_15_Na_6_O_42_] (**e**,**f**). The unit cell is outlined with black color.

The framework of the crystal structure of Cs-exchanged armbrusterite is the same as that of unmodified armbrusterite (Figure 6a,b) [19]. It is primarily composed of complex heteropolyhedral packages. Each package consists of a double silicate tetrahedral layer [Si_9_O_22_]^8–^, which includes 5-, 6-, 7-, and 8-membered tetrahedral rings (Figure 6c,d). Adjacent silicate layers are interconnected by a corrugated octahedral layer with the general formula [Mn^2+^_14_Mn^3+^Na_6_O_42_]^47–^ (Figure 6e,f).

The silicate layer contains nine independent Si positions. The average Si-O bond lengths range from 1.592 to 1.624 Å, which, along with the observed scattering parameters, is consistent with the full occupancy of these positions by Si atoms. The octahedral layer contains two non-equivalent Na positions, Na1 and Na2, in octahedral coordination; the average <Na-O> bond lengths are 2.467 and 2.434 Å, respectively.

Five out of the six octahedral Mn positions have Mn-O bond lengths in the range of 2.158–2.219 Å, while only one position, Mn5, is characterized by an average Mn5-O bond length of 2.067 Å. For the Mn5 position, the calculation of valence sums based on Mn^2+^ yields a result of 2.80 v.u.; similarly, in the original armbrusterite, this position was assigned to Mn^3+^.

**Figure 7:**
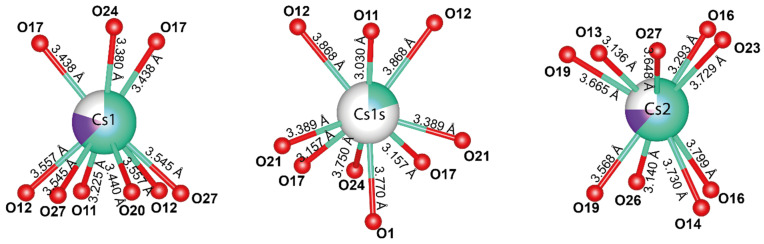
Local coordination of Cs positions in the crystal structure of Cs-exchanged armbrusterite.

The silicate layers contain a two-dimensional network of channels where Cs^+^ atoms occupy three non-equivalent positions: Cs1, Cs1s, and Cs2. The distance between the Cs1 and Cs1s positions is 1.81 Å, implying that only one position can be occupied at a time. The refined occupancy of the Cs1 position is (Cs_0.63_K_0.17_)_0.80_, while the occupancy of the Cs1s position is Cs_0.20_, resulting in a total occupancy of 1. The Cs1 position is coordinated by 9 O atoms with an average bond length of 3.458 Å (Figure 7). The average Cs1s-O bond length is 3.486 Å. The Cs2 position also has a coordination number of 9 and an average bond length of 3.523 Å, with an occupancy of (Cs_0.62_K_0.13_)_0.75_.

The BVS calculation shows slightly underestimated values of 0.39, 0.14, and 0.38 v.u. for the Cs1, Cs1s, and Cs2 positions, respectively. The relatively low valence sum values are associated with the extra framework positioning of the Cs atoms or additional water molecules in the channels that have not been localized.

An analysis of the valence sums on the oxygen atoms revealed that hydroxyl (OH) groups are present in the structure of Cs-substituted armbrusterite. These include O1 (0.79 v.u.), O2 (1.04), O4 (1.11), and O25 (1.04). The occupancy of these positions is consistent with that observed in the original armbrusterite. No additional hydroxyl groups were detected.

The refined crystal chemical formula is (Cs_4.13_K_0.87_)_Σ5.00_Na_6.00_Mn^3+^_1.00_Mn^2+^_14.00_[Si_9_O_22_]_4_(OH)_10.00_.

### 3.3. Infrared Spectroscopy

**Figure 8:**
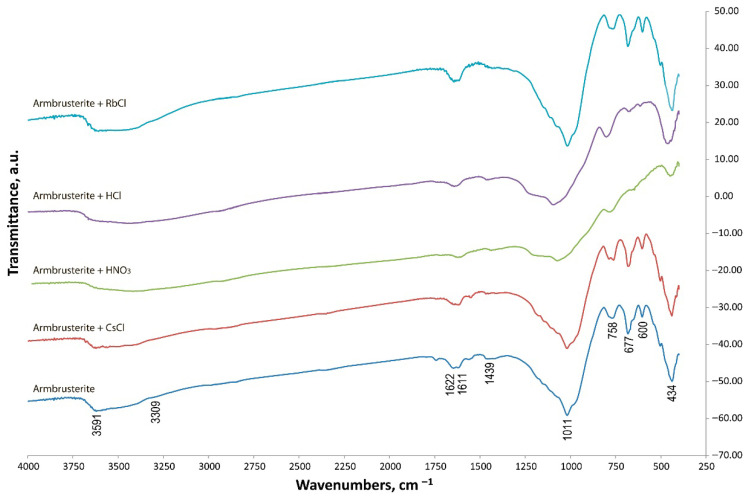
The IR spectra of the unmodified mineral sample and its modifications.

Figure 8 shows the IR spectrum of natural armbrusterite. Absorption bands for spectral assignment were identified according to literature sources [26,27,28].

The IR spectroscopy findings prove the X-ray phase diffraction data and indicate that the samples contain the following groups of atoms. The absorption bands at 3591 and 3309 cm^−1^ are characteristic of ν[OH]; the band at 1611 cm^−1^ [26] is characteristic of δ[HOH] [26]. Mn atoms are present in two configurations: MnO_2_ (1622 cm^−1^) and octahedral MnO (600 cm^−1^) [27]. A similar situation is observed for silicon: the spectra of silicates with tetrahedrally coordinated Si atoms feature absorption bands at 1011 (νasSi–O–Si) and 434 (a mixed vibration δ[SiO] [28] and ν[metal-O] cm^−1^). The band at 1439 cm^−1^ simultaneously corresponds to two vibrations: ν_as_[Si-O-Si] [26] and the stretching modes of terminal SiO- bonds. The vibrations at 758 and 677 cm^−1^ refer to the symmetric stretching modes of the Si–O–Si bridge [26].

One can clearly see that the spectra of natural armbrusterite and the products of treating the mineral with chlorine-containing solutions almost coincide. Therefore, the structure of unmodified armbrusterite has been altered negligibly. The spectrum of the product of treatment with HNO_3_ significantly differs from that of natural armbrusterite, since only non-significant bands corresponding to vibrations Si–O–Si (1050, 758, and 430 cm^−1^) can be detected in this case [26].

### 3.4. Hydrothermal Synthesis of an Armbrusterite Analog

A series of hydrothermal synthesis experiments aiming to produce an analog of the natural mineral, where such variables as weights of the reaction mixture components, potassium sources, and synthesis temperature and duration were varied, revealed that products characterized by good crystallinity degree were obtained in all the cases. The resulting products had both simpler (birnessite) and more complex chemical compositions (serandite, mangani–eckermannite, and the mixed serandite–armbrusterite phase) containing all the elements needed for the formation of the armbrusterite crystal structure (Si, Mn, Na, and K). Table 8 lists the composition of the synthesis reaction mixture and hydrothermal synthesis parameters.

**Figure 9:**
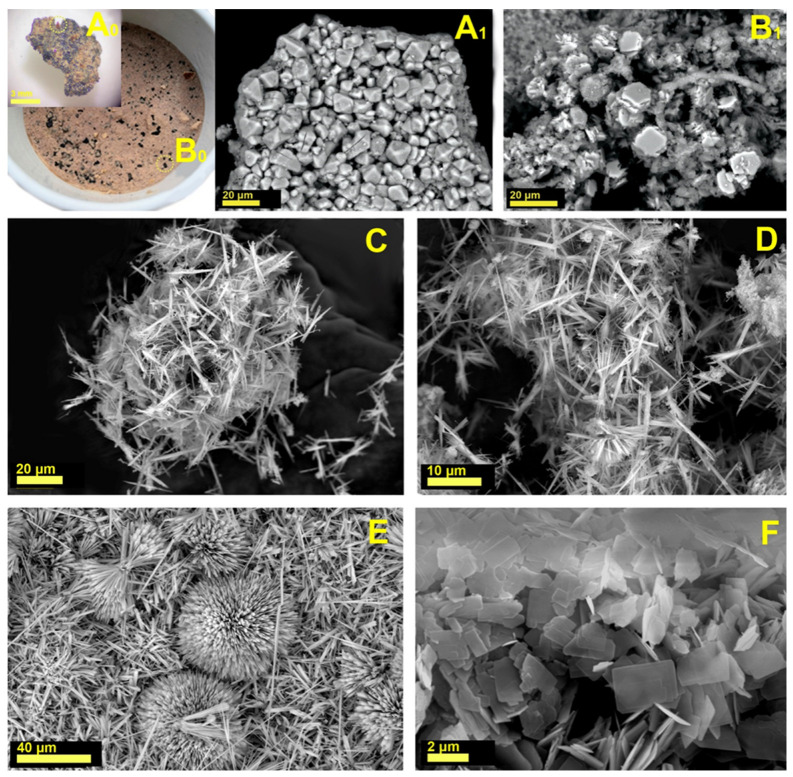
The synthesis products: the multiphase products with admixed crystal-like particles (**A_0_**,**A_1_**); the birnessite phase (**B_0_**,**B_1_**); the single-phase products of serandite (**C**,**D**) and mangani-eckermannite (**E**); a mixture of the serandite and armbrusterite phases (**F**).

All the synthesis products were powdered materials of different colors (black, pink, beige, brown, and violet) having both single-phase and multiphase compositions. Figure 9A_0_,B_0_ shows an example of the color of a multiphase synthesis product.

**Figure 10:**
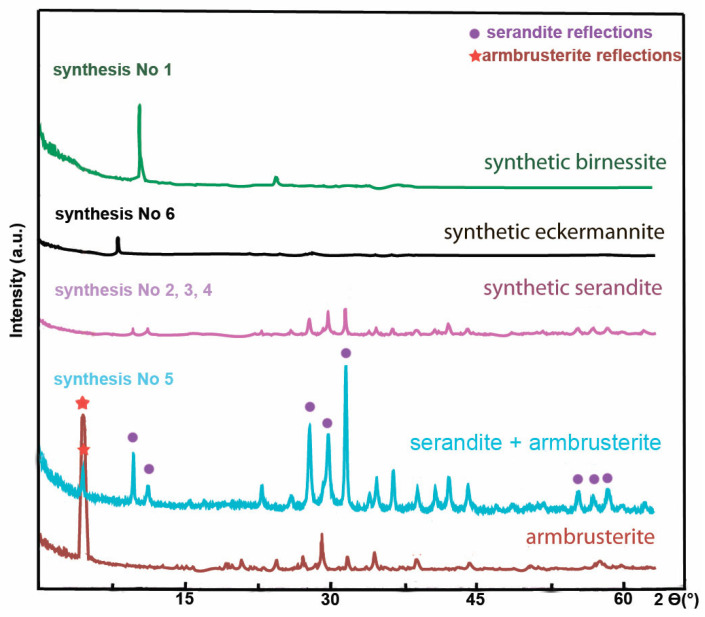
XRD patterns of the synthesis products.

The XRD data demonstrate that multiphase products containing admixed crystal-like particles having no reflections in the XRD patterns (Figure 9A_0_,A_1_ and Figure 10) and the birnessite phase (Figure 9B_0_,B_1_ and Figure 10); the single-phase products of serandite (Figure 9C,D and Figure 10) and mangani-eckermannite (Figure 9E and Figure 10), as well as a powdered mixture of the serandite and armbrusterite phases (Figure 9F and Figure 10), were obtained.

Synthesis experiments revealed that the nature of a potassium source has a significant effect on phase formation of the products. The eckermannite phase and the mixed phase with reflections of armbrusterite (2ϴ range (°): 3–7.5; 31–33; and 37.5–45) and serandite (2ϴ range (°): 7.5–15; 25–30; 52.5–60) were formed in the case of using KOH (Figure 10).

## 4. Discussion

Heterophyllosilicates are rare minerals of complex composition. Synthesizing these compounds is also challenging. The synthesis is usually conducted using platinum capsules (Tuttle bombs) at 380–450 °C. Melt synthesis is another known method for obtaining these compounds [29].

Our study has demonstrated for the first time that these compounds can be synthesized under milder conditions at relatively low temperatures.

The findings demonstrate that phases with a more complex chemical composition in the order (birnessite; serandite) → mangani-eckermannite → a mixture of phases with the peaks characteristic of armbrusterite and serandite are synthesized at elevated treatment temperatures. Nevertheless, pressure and exposure duration of the reaction mixture need to be adjusted to obtain the armbrusterite monophase.

According to the single-crystal analysis data, the crystal structure of Arm:Cs mainly includes Cs^+^ cations without H_2_O molecules. This fact may be attributed to the specific features of the orientational ion–dipole interaction between molecules of a strong polar solvent (water molecules) around cesium ions. The Cs^+^ ion possesses a large effective ionic radius and, consequently, a lower surface charge density compared to other alkali cations, resulting in weaker hydration and reduced affinity for water molecules. One should bear in mind that cesium cations have an appreciably small hydration number (1–2) compared to Na^+^ cations (4–5) [30]. Hence, cesium cations are characterized by weaker attraction of water molecules compared to sodium cations. The charge of the mineral framework in the interlayer space also makes its contribution, being responsible for the fact that Cs^+^ cations preferentially bind to oxygen atoms rather than to water molecules. In their turn, water molecules are not completely immobilized and restrained. They can switch positions with molecules in the solution as it contacts the crystal. Hence, the nature of hydration of water ions is also affected by the average time during which a water molecule can stay near the cation. For Cs^+^, the average reorientation time is as short as 10^−10^–10^−9^ s [30].

Extra-framework cations in the structure of armbrusterite reside within the framework channels. Channel I, extending along [010], is characterized by a 16-cross-section and an effective diameter (for non-isometric channels with elliptical cross-sections, according to IUPAC nomenclature, the lengths of the major and minor axes minus the sum of the two ionic radii of O, 2.7 Å) [31], equal to 14.52 × 3.53 Å^2^. Channel II, parallel to the [100] direction, has an octagonal cross-section and an effective diameter of 4.84 × 3.21 Å^2^. Along the [100] direction, there is channel III, which has a 16-cross-section and an effective diameter of 13.19 × 3.04 Å^2^. The overall negative charge of the framework (5-) is compensated for by cations at positions Cs1, Cs1s, and Cs2, with the total refined occupancy of (Cs_4.13_K_0.87_)_Σ5.00_.

**Figure 11:**
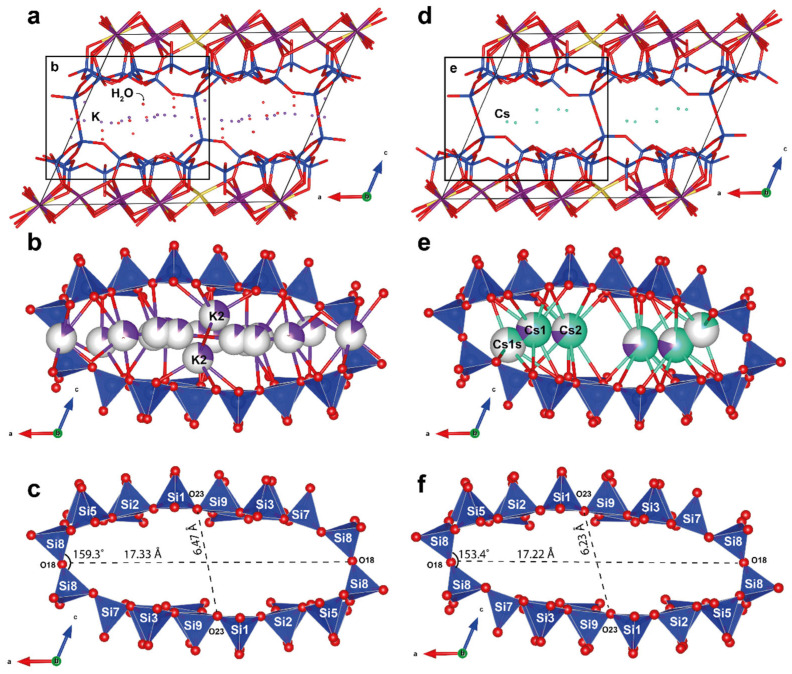
Projection of crystal structures along the b-axis of armbrusterite (**a**) and the Cs-substituted form of armbrusterite (**d**); cation arrangements in channels I in armbrusterite (**b**) and the Cs-substituted form of armbrusterite (**e**); and geometrical characterization of the channels of armbrusterite (**c**) and the Cs-substituted form of armbrusterite (**f**).

The structural topologies of the original armbrusterite and the Cs-substituted armbrusterite are identical. The difference in the unit cell volumes between armbrusterite and its Cs-substituted form is 206.3 Å^3^. The original compound has a larger volume, possibly due to the presence of additional four water molecules and the specific arrangement of K within the structure of original armbrusterite. Since the atomic radius of K^+^ (1.69 Å) is significantly smaller than that of Cs^+^ (1.92 Å), two K_2_ polyhedra (Figure 6b) occupy the centers of the largest channels I (Figure 11a,d) at different *z* levels [32]. In contrast, the most populated Cs1 and Cs2 polyhedra are situated at the same *z* level (Figure 11e). The ordered arrangement of Cs within the channels leads to reduction of the dihedral angle ∠Si8O18Si8 from 159.3° in armbrusterite to 153.4° in Cs-substituted armbrusterite. This deformation decreases the short diagonal of the channel from 6.47 Å in armbrusterite to 6.23 Å in Cs-substituted armbrusterite (Figure 11a,c), while the long diagonal remains relatively unchanged (17.33 vs. 17.22 Å). Thus, the migration of extra-framework cations during ion exchange affects the overall deformation of the crystal structure framework. Similar phenomena have previously been observed in minerals from the Kola alkaline province, specifically ivanyukite [33] and sitinakite [34,35].

## 5. Conclusions

Our study has proved that the extra-framework potassium cations can be entirely eliminated from the structure of natural armbrusterite by treating crystals of the mineral with diluted acidic solutions. K^+^ cations are almost completely exchanged for Cs^+^ cations and occupy three independent positions in the crystal structure pores. Water molecules leave the crystal structure. The incorporation of Cs into armbrusterite is accompanied by complex mechanisms of cooperative crystal chemical adaptation in which the silicate framework geometry is affected by arrangement of extra-framework cations.

It has been experimentally verified for the solutions of single-component salts (containing a Cs, Rb, Sr, or Cd cation) that the mineral exhibits good sorption properties with respect to monovalent cations of Cs^+^ and Rb^+^ (Cs_2_O, 14.18 wt.%; Rb_2_O, 5.03 wt.%). This fact is also proved by analyzing the sorption properties of the mineral treated with a multicomponent solution. When the mineral contacts a solution of complex chemical composition, Cs^+^ cations are selectively extracted from the solution (Cs_2_O, up to 8 wt.%).

The selected conditions of hydrothermal synthesis of the mineral analog demonstrate that it is possible to produce well-decrystallized Mn-Si compounds, but the duration of reaction mixture exposure needs to be further optimized, and synthesis temperature probably needs to be increased.

## Figures and Tables

**Table 1:** Crystal data and structure refinement for Cs-exchanged armbrusterite.

Source	This Work	[19]
Sample	Armbrusterite Cs-exchanged	Armbrusterite
Temperature/K	293(2)	
Crystal system	monoclinic	
Space group	*C*2/*m*	
entry 5	data	
*a*/Å	17.226(4)	17.333(2)
*b*/Å	23.437(3)	23.539(3)
*c*/Å	12.926(2)	13.4895(17)
β/°	113.687(15)	115.069(9)
Volume/Å^3^	4779.1(16)	4985.4(11)
*Z*	2	
ρ_calc_g/cm^3^	2.866	2.53
μ/mm^−1^	4.175	
F(000)	3941.0	
Crystal size/mm^3^	0.12 × 0.05 × 0.05	0.22 × 0.16 × 0.04
Radiation	Mo Kα (λ = 0.71073)	
2Θ range for data collection/°	6.746 to 50.994	
Index ranges	−20 ≤ h ≤ 19, −27 ≤ k ≤ 27, −15 ≤ l ≤ 13	
Reflections collected	7319	5414
Independent reflections	4019 [Rint = 0.0961, Rsigma = 0.1222]	3960
Data/restraints/parameters	4019/0/382	3960
Goodness-of-fit on F2	1.226	1.069
Final R indices [I ≥ 2σ (I)]	R1 = 0.1304, wR2 = 0.3301	R1 = 0.086, wR2 = 0.212
Final R indices [all data]	R1 = 0.1871, wR2 = 0.3696	
Largest diff. peak/hole/e Å^−3^	2.98/−2.71	

**Table 2:** Atomic coordinates and occupancies (Å^2^) in the crystal structure of Arm:Cs.

Site	s.o.f.	x	y	z	Uiso	BVS *
Mn1	Mn^2+^	½	0.56959(17)	0	0.0276(10)	2.29
Mn2	Mn^2+^	½	0.71179(16)	0	0.0249(10)	2.05
Mn3	Mn^2+^	0.82484(17)	0.57176(12)	−0.0398(3)	0.0268(8)	1.98
Mn4	Mn^2+^	0.6527(2)	½	−0.0434(4)	0.0293(11)	1.93
Mn5	Mn^3+^	0	½	0	0.0241(13)	2.80
Mn6	Mn^2+^	0.67229(17)	0.78510(12)	0.0178(3)	0.0277(8)	1.98
Si1	Si^4+^	0.7835(3)	0.5639(2)	0.1859(5)	0.0252(12)	4.27
Si2	Si^4+^	0.6433(3)	0.6321(2)	0.2145(5)	0.0261(12)	4.23
Si3	Si^4+^	0.0970(3)	0.5641(2)	0.2370(5)	0.0259(12)	4.09
Si4	Si^4+^	0.6377(3)	0.7554(2)	0.2431(5)	0.0243(12)	4.06
Si5	Si^4+^	0.5087(3)	0.5646(2)	0.2555(5)	0.0255(12)	4.15
Si6	Si^4+^	0.4653(3)	0.7456(2)	0.2359(5)	0.0266(12)	4.13
Si7	Si^4+^	0.2455(3)	0.6417(2)	0.2798(5)	0.0263(12)	4.10
Si8	Si^4+^	0.4341(3)	0.6452(2)	0.3748(5)	0.0272(13)	4.39
Si9	Si^4+^	0.9358(3)	0.6313(2)	0.2064(5)	0.0269(13)	4.22
Na1	Na^+^	0.8459(5)	0.8560(3)	0.0482(7)	0.0330(18)	1.06
Na2	Na^+^	0	0.6493(5)	0	0.039(3)	1.14
Cs1	Cs^+^_0.63_K^+^_0.17_	0.7389(2)	½	0.4618(4)	0.081(2)	0.39
Cs1S	Cs^+^_0.20_	0.6719(10)	½	0.5391(12)	0.070(7)	0.14
Cs2	Cs^+^_0.62_K^+^_0.13_	0.6353(2)	0.8125(2)	0.5336(3)	0.115(2)	0.39
O1	OH^−^	0.7361(11)	½	−0.145(2)	0.036(3)	0.79
O2	OH^−^	−0.0910(11)	½	0.0626(17)	0.025(4)	1.04
O3	O^2−^	0.7422(7)	0.5665(4)	0.0492(13)	0.027(3)	1.95
O4	OH^−^	0.4134(10)	½	−0.0730(17)	0.026(4)	1.11
O5	O^2−^	0.9015(6)	0.6363(4)	0.0748(13)	0.026(3)	2.08
O6	O^2−^	0.2356(8)	0.6421(5)	0.1534(12)	0.030(3)	1.82
O7	O^2−^	0.5936(7)	0.7697(5)	0.1110(12)	0.029(3)	1.96
O8	O^2−^	0.4455(8)	0.5657(5)	0.1273(13)	0.030(3)	1.98
O9	O^2−^	0.5832(8)	0.6369(5)	0.0844(13)	0.031(3)	1.99
O10	O^2−^	0.0748(7)	0.5635(5)	0.1066(12)	0.028(3)	1.99
O11	O^2−^	0.5399(11)	½	0.298(2)	0.039(5)	2.12
O12	O^2−^	0.5945(8)	0.6022(6)	0.2825(12)	0.034(3)	2.10
O13	O^2−^	0.6832(8)	0.6934(5)	0.2709(13)	0.032(3)	2.09
O14	O^2−^	0.0324(8)	0.6051(5)	0.2660(12)	0.031(3)	2.00
O15	O^2−^	0.4292(7)	0.7302(6)	0.1038(13)	0.032(3)	1.91
O16	O^2−^	0.9347(8)	0.6922(5)	0.2639(13)	0.030(3)	2.09
O17	O^2−^	0.1939(8)	0.5893(5)	0.3079(13)	0.031(3)	1.97
O18	O^2−^	½	0.6608(10)	½	0.049(6)	2.13
O19	O^2−^	0.2131(8)	0.7001(5)	0.3171(12)	0.028(3)	2.03
O20	O^2−^	0.7923(12)	½	0.2327(18)	0.035(5)	2.20
O21	O^2−^	0.3432(7)	0.6344(6)	0.3742(12)	0.033(3)	2.08
O22	O^2−^	0.4306(8)	0.6991(6)	0.2969(14)	0.039(4)	2.19
O23	O^2−^	0.8775(8)	0.5902(6)	0.2454(14)	0.038(4)	2.11
O24	O^2−^	0.0943(12)	½	0.2848(17)	0.030(4)	2.02
O25	OH^−^	0.7512(8)	0.7891(5)	−0.0729(14)	0.036(3)	1.04
O26	O^2−^	0.5679(7)	0.7504(5)	0.2986(13)	0.029(3)	2.09
O27	O^2−^	0.7273(9)	0.5942(6)	0.2415(13)	0.040(4)	2.21
O28	O^2−^	0.4644(8)	0.5885(6)	0.3363(14)	0.040(4)	2.16

* Refined formula: (Cs_4.13_K_0.87_)_Σ5.00_Na_6.00_Mn^3+^_1.00_Mn^2+^_14.00_[Si_9_O_22_]_4_(OH)_10.00_.

**Table 3:** Selected bond lengths (Å) in the crystal structure of Arm:Cs.

Mn1-O4	2.155(11) 2×	Na2-O5	2.285(12) 2×	Si3-O10	1.573(16)
Mn1-O8	2.199(14) 2×	Na2-O7	2.531(15) 2×	Si3-O14	1.623(12)
Mn1-O9	2.115(13) 2×	Na2-O10	2.485(15) 2×	Si3-O17	1.657(14)
<Mn1-O>	2.156	<Na2-O>	2.434	Si3-O24	1.633(9)
				<Si3-O>	1.622
Mn2-O7	2.154(13) 2×	Cs1-O11	3.225(19)		
Mn2-O9	2.251(13) 2×	Cs1-O12	3.557(15) 2×	Si4-O7	1.600(15)
Mn2-O9	2.187(13) 2×	Cs1-O17	3.438(14) 2×	Si4-O13	1.622(13)
<Mn2-O>	2.197	Cs1-O20	3.44(2)	Si4-O19	1.640(13)
		Cs1-O24	3.38(2)	Si4-O26	1.633(13)
Mn3-O1	2.313(15)	Cs1-O27	3.545(16) 2×	<Si4-O>	1.624
Mn3-O2	2.267(13)	<Cs1-O>	3.458		
Mn3-O3	2.164(13)			Si5-O8	1.576(16)
Mn3-O5	2.157(12)	Cs1S-O1	3.77(3)	Si5-O11	1.626(9)
Mn3-O6	2.174(13)	Cs1S-O11	3.03(3)	Si5-O12	1.634(13)
Mn3-O10	2.229(12)	Cs1S-O17	3.157(16) 2×	Si5-O28	1.620(14)
<Mn3-O>	2.209	Cs1S-O21	3.389(15) 2×	<Si5-O>	1.614
		Cs1S-O24	3.75(2)		
Mn4-O1	2.306(19)	Cs1S-O12	3.868(19) 2×	Si6-O15	1.607(16)
Mn4-O3	2.180(12) 2×	<Cs1s-O>	3.486	Si6-O16	1.639(13)
Mn4-O4	2.223(19)			Si6-O22	1.594(14)
Mn4-O8	2.221(13) 2×	Cs2-O13	3.136(15)	Si6-O26	1.626(12)
<Mn4-O>	2.222	Cs2-O14	3.730(14)	<Si6-O>	1.617
		Cs2-O16	3.293(14)		
Mn5-O2	2.033(17) 2×	Cs2-O16	3.799(15)	Si7-O6	1.574(15)
Mn5-O10	2.084(13) 4×	Cs2-O19	3.568(14)	Si7-O17	1.639(13)
<Mn5-O>	2.067	Cs2-O19	3.665(13)	Si7-O19	1.624(12)
		Cs2-O23	3.729(16)	Si7-O21	1.643(13)
Mn6-O5	2.285(11)	Cs2-O26	3.140(15)	<Si7-O>	1.620
Mn6-O6	2.375(13)	Cs2-O27	3.648(16)		
Mn6-O7	2.176(12)	<Cs2-O>	3.523	Si8-O18	1.601(8)
Mn6-O15	2.230(14)			Si8-O21	1.583(12)
Mn6-O25	2.123(13) 2×	Si1-O3	1.619(16)	Si8-O22	1.603(15)
<Mn6-O>	2.219	Si1-O20	1.598(9)	Si8-O28	1.579(14)
		Si1-O23	1.610(13)	<Si8-O>	1.592
Na1-O3	2.377(14)	Si1-O27	1.588(14)		
Na1-O6	2.744(16)	<Si1-O>	1.604	Si9-O5	1.565(16)
Na1-O8	2.440(14)			Si9-O14	1.645(13)
Na1-O9	2.478(16)	Si2-O9	1.586(16)	Si9-O16	1.614(13)
Na1-O15	2.415(15)	Si2-O12	1.602(13)	Si9-O23	1.612(13)
Na1-O25	2.350(16)	Si2-O13	1.632(13)	<Si9-O>	1.609
<Na1-O>	2.467	Si2-O27	1.612(15)		
		<Si2-O>	1.608		

**Table 4:** Anisotropic parameters of atomic displacements (Å^2^) in the crystal structure of Arm:Cs.

Atom	*U*_11_	*U*_22_	*U*_33_	*U*_23_	*U*_13_	*U*_12_
Mn1	0.028(2)	0.029(2)	0.037(3)	0.000	0.0236(19)	0.000
Mn2	0.0250(19)	0.026(2)	0.032(3)	0.000	0.0207(18)	0.000
Mn3	0.0251(15)	0.0287(15)	0.036(2)	0.0009(13)	0.0220(13)	0.0005(11)
Mn4	0.027(2)	0.028(2)	0.042(3)	0.000	0.025(2)	0.000
Mn5	0.024(3)	0.026(3)	0.030(4)	0.000	0.019(3)	0.000
Mn6	0.0263(15)	0.0286(16)	0.038(2)	0.0004(13)	0.0225(14)	0.0015(11)
Si1	0.027(2)	0.022(2)	0.038(3)	−0.001(2)	0.024(2)	0.0006(19)
Si2	0.026(3)	0.030(3)	0.033(3)	−0.001(2)	0.022(2)	0.001(2)
Si3	0.025(2)	0.032(3)	0.031(3)	0.000(2)	0.022(2)	0.001(2)
Si4	0.021(2)	0.029(3)	0.029(3)	−0.001(2)	0.017(2)	−0.0010(19)
Si5	0.029(3)	0.024(2)	0.036(3)	0.000(2)	0.025(2)	0.0015(19)
Si6	0.028(3)	0.030(3)	0.030(3)	0.003(2)	0.021(2)	−0.002(2)
Si7	0.021(2)	0.029(3)	0.036(3)	0.001(2)	0.018(2)	0.0015(19)
Si8	0.026(3)	0.026(3)	0.040(4)	0.002(2)	0.025(2)	0.001(2)
Si9	0.023(2)	0.025(3)	0.043(4)	0.002(2)	0.024(2)	−0.0034(19)
Na1	0.034(4)	0.032(4)	0.038(5)	0.000(3)	0.020(4)	−0.004(3)
Na2	0.034(6)	0.043(6)	0.050(9)	0.000	0.028(6)	0.000
Cs1	0.059(3)	0.129(4)	0.049(3)	0.000	0.0170(19)	0.000
Cs1S	0.088(11)	0.048(8)	0.039(9)	0.000	−0.011(7)	0.000
Cs2	0.082(3)	0.212(6)	0.054(2)	−0.037(3)	0.0295(18)	−0.006(3)
O1	0.022(5)	0.033(6)	0.065(9)	0.000	0.033(6)	0.000
O2	0.031(9)	0.018(8)	0.032(12)	0.000	0.018(9)	0.000
O3	0.014(5)	0.012(5)	0.060(10)	0.002(5)	0.021(6)	−0.003(4)
O4	0.020(8)	0.021(9)	0.034(12)	0.000	0.010(8)	0.000
O5	0.007(5)	0.011(5)	0.064(11)	0.003(5)	0.017(6)	0.000(4)
O6	0.031(7)	0.037(7)	0.024(8)	0.003(6)	0.012(6)	−0.002(5)
O7	0.019(6)	0.033(7)	0.045(9)	0.001(6)	0.024(6)	−0.005(5)
O8	0.039(7)	0.012(5)	0.046(10)	0.002(5)	0.026(7)	−0.003(5)
O9	0.040(7)	0.015(6)	0.051(10)	0.004(6)	0.033(7)	−0.003(5)
O10	0.009(5)	0.039(7)	0.035(9)	0.001(6)	0.009(5)	0.004(5)
O11	0.026(9)	0.046(12)	0.047(15)	0.000	0.017(10)	0.000
O12	0.028(7)	0.048(8)	0.036(9)	0.000(7)	0.022(6)	−0.011(6)
O13	0.034(7)	0.031(7)	0.049(10)	−0.007(6)	0.035(7)	−0.007(5)
O14	0.031(7)	0.032(7)	0.041(9)	−0.004(6)	0.027(6)	0.002(5)
O15	0.023(6)	0.042(7)	0.044(9)	0.000(7)	0.028(6)	0.002(5)
O16	0.030(6)	0.030(7)	0.046(9)	−0.004(6)	0.033(7)	−0.003(5)
O17	0.030(7)	0.031(7)	0.046(10)	0.004(6)	0.030(7)	0.005(5)
O18	0.035(11)	0.077(16)	0.042(15)	0.000	0.023(11)	0.000
O19	0.037(7)	0.017(6)	0.036(9)	0.001(5)	0.022(6)	0.010(5)
O20	0.049(11)	0.035(10)	0.035(13)	0.000	0.033(10)	0.000
O21	0.021(6)	0.049(8)	0.036(9)	0.004(6)	0.019(6)	0.010(5)
O22	0.030(7)	0.042(8)	0.056(11)	0.009(7)	0.028(7)	−0.001(6)
O23	0.029(7)	0.041(8)	0.054(11)	0.002(7)	0.029(7)	−0.006(6)
O24	0.050(11)	0.020(9)	0.035(13)	0.000	0.034(10)	0.000
O25	0.022(5)	0.033(6)	0.065(9)	0.000	0.033(6)	0.000
O26	0.017(6)	0.035(7)	0.041(9)	−0.005(6)	0.018(6)	−0.001(5)
O27	0.049(8)	0.046(8)	0.038(10)	−0.002(7)	0.032(7)	0.005(7)
O28	0.039(8)	0.036(7)	0.066(12)	−0.009(7)	0.043(8)	0.003(6)

**Table 5:** The chemical composition of the polished samples of the unmodified mineral (Arm) and the samples treated with 1 M solutions of acids (Arm: HNO_3_; Arm: HCl).

Oxide	Content, %		
	Arm	Arm:HNO_3_	Arm:HCl
Na_2_O	3.86	3.77	1.17
K_2_O	9.57	1.87	0.73
SiO_2_	51.12	70.20	75.87
MnO	23.41	16.57	13.54
Mn_2_O_3_	2.07 *	2.07 *	2.07 *
CaO	0.19	-	0.13
TiO_2_	0.14	0.12	0.15
FeO	0.70	0.66	0.42
ZnO	0.15	-	-
Al_2_O_3_	0.11	-	-
MgO	0.17	-	-
H_2_O **	8.51	4.74	5.92
Total	100	100	100
Formula coefficients on the basis 36 Si atoms			
Na (a.p.f.u.)	5.27	3.75	1.08
K	8.60	1.22	0.44
Si	36.00	36.00	36.00
Mn^2+^	13.96	7.20	5.44
Mn^3+^	1.11	0.81	0.75
Ti	0.07	0.05	0.05
Ca	0.14	-	0.07
Al	0.09	-	-
Fe	0.41	0.28	0.17
Zn	0.08	-	-
H	7.65	3.10	3.59
Σ_cations_	73.38	52.41	47.59

* calculated on the basis of ideal formula (1Mn 3+ apfu). ** calculated from the lack of the sum of the analysis.

**Table 6:** Powder diffraction data: the strongest reflections of armbrusterite (Arm) and its ion-exchange forms: Arm:Cs, Arm:Rb, Arm:Cd and Arm:mix.

Arm			Arm:Cs			Arm:Rb			Arm:Mix		
I_meas_	2θ (°)	d (Å)	I_meas_	2θ (°)	d (Å)	I_meas_	2θ (°)	d (Å)	I_meas_	2θ (°)	d (Å)
100	7.20	12.27	100	7.39	11.95	100	7.20	12.27	100	7.30	12.10
2	21.71	4.090	5	25.54	3.484	3	20.41	4.347	6	25.33	3.513
2	25.00	3.558	4	27.67	3.221	2	21.75	4.083	7	27.73	3.215
2	27.39	3.254	16	28.32	3.149	4	27.42	3.250	11	27.96	3.188
9	29.09	3.067	5	28.97	3.080	17	29.14	3.062	34	29.55	3.020
1	54.91	1.671	30	29.94	2.982	2	34.18	2.620	5	55.19	1.633

**Table 7:** The chemical composition of the sections of polished samples of unmodified armbrusterite (Arm) and those treated with solutions of salts (CsCl, RbCl, CdCl_2_, and a solution of mixed composition). Analyses with exchangeable cation content below 0.35 wt% are not presented due to the impracticality of considering such samples as promising sorbents.

Oxide	Content, %				
	Arm	Arm:Cs	Arm:Rb	Arm:Cd	Arm:Mixed Solution
Na_2_O	3.86	3.86	2.94	4.33	2.42
K_2_O	9.57	0.33	4.00	7.18	1.15
SiO_2_	51.12	53.70	52.25	52.17	53.31
MnO	23.41	23.71	24.00	23.40	25.42
Mn_2_O_3_	2.07 *	2.07 *	2.07 *	2.07 *	2.07 *
Al_2_O_3_	0.11	-	0.16	0.13	0.20
CaO	0.19	0.16	0.18	0.20	0.23
MgO	0.17	-	0.16	0.15	0.19
FeO	0.70	0.67	0.72	0.69	0.76
TiO_2_	0.14	-	0.10	0.09	0.15
ZnO	0.15	-	0.18	0.21	-
Cs_2_O	-	14.18	-	-	8.39
Rb_2_O	-	-	5.03	-	0.52
SrO	-	-	-	-	0.34
CdO	-	-	-	0.35	0.10
H_2_O **	8.88	1.8	8.93	9.03	5.51
Total	91.12	98.2	91.07	90.97	94.49
Formula coefficients on the basis 36 Si atoms					
Na (a.p.f.u.)	5.27	5.02	3.93	5.79	3.17
K	8.60	0.28	3.52	6.32	1.0
Si	36.00	36.00	36.00	36.00	36.00
Mn^2+^	13.96	13.46	14.00	13.68	14.00
Mn^3+^	1.11	1.06	1.09	1.09	1.09
Ti	0.07	-	0.05	0.05	0.05
Ca	0.14	0.12	0.13	0.15	0.13
Al	0.09	-	0.13	0.11	0.13
Fe	0.41	0.38	0.42	0.40	0.42
Zn	0.08	-	-	0.11	-
Cs	-	4.05	-	-	2.42
Rb	-	-	2.23	-	0.23
Sr	-	-	-	-	-
Cd	-	-	-	0.11	0.03
H	7.65	4.22	5.57	16.27	4.88
Σ_cations_	73.38	64.59	67.07	80.08	63.55

* Calculated on the basis of ideal formula (1Mn 3+ apfu). ** calculated from the lack of the sum of the analysis.

**Table 8:** Sample weights and parameters of hydrothermal synthesis of an analog of the natural mineral.

Synthesis No.	Weights of Reagents, g					T, °C	Time, h	Result
	Na_2_SiO_3_∙5H_2_O	MnSO_4_∙5H_2_O	KOH	KCl	H_2_O			
1	7.73	3.78	0.75	-	35	200	96	Birnessite
2	13.8	3.37	0.37	0.5	33	200	96	Serandite
3	3,45	0.84	0,46	-	15	270	96	Serandite
4	1.93	0.47	0.20	-	12	200	240	Serandite
5	1.93	0.47	0.20	-	15	270	96	Serandite + armbrusterite
6	1.93	0.47	0.20	-	12	270	168	Mangani-eckermannite

## Data Availability

The original contributions presented in this study are included in the article. Further inquiries can be directed to the corresponding author.
